# Global rainfall erosivity assessment based on high-temporal resolution rainfall records

**DOI:** 10.1038/s41598-017-04282-8

**Published:** 2017-06-23

**Authors:** Panos Panagos, Pasquale Borrelli, Katrin Meusburger, Bofu Yu, Andreas Klik, Kyoung Jae Lim, Jae E. Yang, Jinren Ni, Chiyuan Miao, Nabansu Chattopadhyay, Seyed Hamidreza Sadeghi, Zeinab Hazbavi, Mohsen Zabihi, Gennady A. Larionov, Sergey F. Krasnov, Andrey V. Gorobets, Yoav Levi, Gunay Erpul, Christian Birkel, Natalia Hoyos, Victoria Naipal, Paulo Tarso S. Oliveira, Carlos A. Bonilla, Mohamed Meddi, Werner Nel, Hassan Al Dashti, Martino Boni, Nazzareno Diodato, Kristof Van Oost, Mark Nearing, Cristiano Ballabio

**Affiliations:** 1 European Commission, Joint Research Centre, I-21027 Ispra (VA), Italy; 2 Environmental Geosciences, University of Basel, Basel, Switzerland; 3 School of Engineering, Griffith University, Nathan, Australia; 4 BOKU, University of Natural Resources and Life Sciences, Vienna, Austria; 5 Kangwon National University, Chuncheon-si, Gangwon-do, South Korea; 6 College of Environmental Sciences and Engineering, Peking University, Beijing, P.R. China; 7 College of Global Change and Earth System Science, Beijing Normal University, Beijing, P.R. China; 8 India Meteorological Department, Pune, India; 9 Faculty of Natural Resources, Tarbiat Modares University, Jalal, Iran; 10 Faculty of Geography, Lomonosov Moscow State University, Moscow, Russian Federation; 11 Israel Meteorological Service, Beit Dagan, Israel; 12 Faculty of Agriculture - Soil Science Departement, Ankara University, Ankara, Turkey; 13 University of Costa Rica, San Jose, Costa Rica; 14 Universidad del Norte, Barranquilla, Colombia; 15 Laboratoire des Sciences du Climat et de l′Environnement, IPSL-LSCE, Gif sur Yvette, France; 16 Federal University of Mato Grosso do Sul, Campo Grande, Brazil; 17 Departamento de Ingeniería Hidráulica y Ambiental, Pontificia Universidad Católica de Chile, Región Metropolitana, Chile; 18 Ecole Nationale Supérieure d’Hydraulique de Blida, Soumaâ, Algeria; 19 Department of Geography and Environmental Science, University of Fort Hare, Alice, South Africa; 20 Department of Meteorology, Kuwait, Kuwait; 21 Met European Research Observatory, Benevento, Italy; 22 Université Catholique de Louvain, Louvain-la-Neuve, Belgium; 23 USDA-ARS, Southwest Watershed Research Center, Tucson, AZ USA

## Abstract

The exposure of the Earth’s surface to the energetic input of rainfall is one of the key factors controlling water erosion. While water erosion is identified as the most serious cause of soil degradation globally, global patterns of rainfall erosivity remain poorly quantified and estimates have large uncertainties. This hampers the implementation of effective soil degradation mitigation and restoration strategies. Quantifying rainfall erosivity is challenging as it requires high temporal resolution(<30 min) and high fidelity rainfall recordings. We present the results of an extensive global data collection effort whereby we estimated rainfall erosivity for 3,625 stations covering 63 countries. This first ever Global Rainfall Erosivity Database was used to develop a global erosivity map at 30 arc-seconds(~1 km) based on a Gaussian Process Regression(GPR). Globally, the mean rainfall erosivity was estimated to be 2,190 MJ mm ha^−1^ h^−1^ yr^−1^, with the highest values in South America and the Caribbean countries, Central east Africa and South east Asia. The lowest values are mainly found in Canada, the Russian Federation, Northern Europe, Northern Africa and the Middle East. The tropical climate zone has the highest mean rainfall erosivity followed by the temperate whereas the lowest mean was estimated in the cold climate zone.

## Introduction

Given the growing concerns about climate change, climatic data is particularly important for the scientific community and society in general, as decisions of individuals, business and governments are dependent on available meteorological data^1^. At present, a large number of the large-scale precipitation datasets are publicly available, although formats and completeness of the records vary widely^2^. Heavy rainfall and extreme events are of major importance for climate change, economy and society^3^. However, extreme events are typically rare events of short duration and in many regions of the world only limited observational data of sufficient temporal resolution is available to capture them^4^. High temporal resolution rainfall measurements are important in this context, but also have been recognised to be very useful for urban drainage models^5^, climate change modelling^6^, cropping patterns and crop production^7^.

Further, the patterns of heavy and violent rainfall, as captured by the rainfall erosivity factor, influence hydrological and erosive processes and as such are essential for the definition of soil and water conservation practices in the adaptation of agriculture to climate change^8^. Rainfall erosivity is one of the most important input parameters for describing erosive processes and proposing conservation measures by using soil erosion prediction models. Since soil erosion is difficult to measure at large scales, models are required for estimating soil loss by water erosion at regional, national and continental scales. Large scale and global model predictions are of utmost importance, since soil erosion is, in addition to soil sealing, the major threat to soil sustainability and consequently to water- and food security.

As a consequence, recent developments in the soil modelling and climate change communities aim at addressing major scientific gaps in describing key soil processes such as water erosion^9^, based on updated global datasets. Nonetheless, policy initiatives such as the United Nations Convention to Combat Desertification (UNCCD) for zero net land degradation^10^, the Intergovernmental Science-Policy Platform on Biodiversity and Ecosystem Services (IPBES), the Land Degradation and Restoration Assessment^11^, and the Food Agricultural Organisation (FAO) World Soil Resources^12^ still remarked the lack of an updated scientific dataset on global soil erosion.

A vital component of such a global soil erosion map is a spatial assessment of rainfall erosivity. However, methods that estimate erosivity based on annual rainfall data are problematic and highly biased, since rainfall intensity is typically not considered^13^. In order to include rainfall intensity on the calculation of rainfall erosivity, it is necessary to have high temporal resolution rainfall data for long time periods. In this study, we aim to tackle the challenging task of compiling and processing the first global erosivity dataset from long-term high-resolution rainfall data using sub-hourly and hourly pluviographic records. We have used this global erosivity station dataset combined with a set of exhaustive secondary environment variables to generate a global rainfall erosivity map in order to improve our understanding of the global patterns of high intensity rainfall events.

## Results

### Global Rainfall Erosivity Database - GloREDa

At global scale, this is the first time ever that an erosivity database of such dimension is compiled. The Global Rainfall Erosivity Database, named hereafter as **GloREDa**, contains erosivity values estimated as R-factors (refer to the method section) from 3,625 stations distributed in 63 countries worldwide. This is the result of an extensive data collection of high temporal resolution rainfall data from the maximum possible number of countries in order to have a representative sample across different climatic and geographic gradients. GloREDa has three components, which are described in the methods section: a) the Rainfall Erosivity database at European Scale (REDES)^14^ b) 1,865 stations from 23 countries outside Europe and c) 85 stations collected from a literature review.

**Figure 1:**
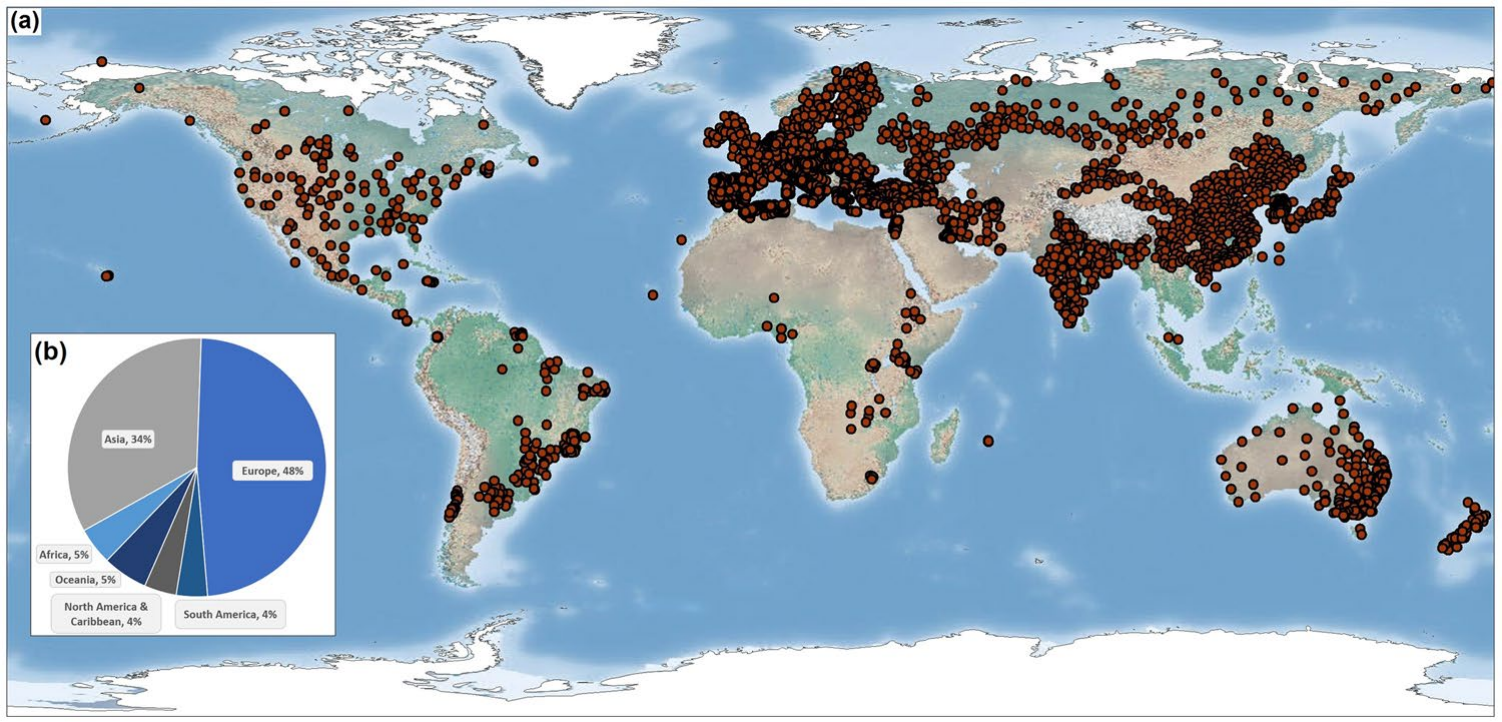
(**a**) Global distribution of rainfall erosivity stations (red dots) compiled in the Global Rainfall Erosivity Database (GloREDa); (**b**) Distribution of rainfall erosivity stations by continent. Maps generated with ESRI ArcGIS ver. 10.4 (http://www.esri.com).

**Figure 2:**
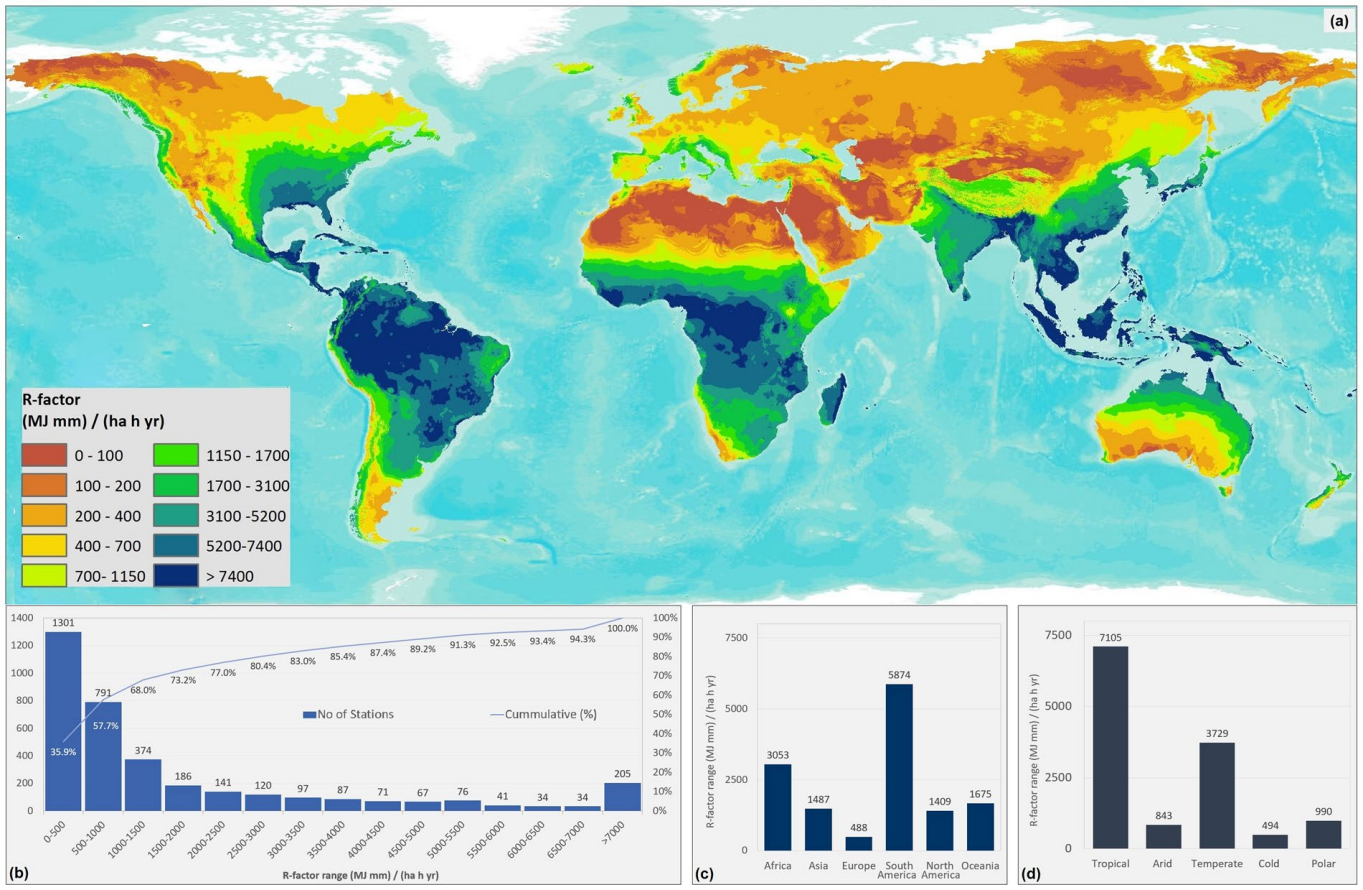
(**a**) Global Rainfall Erosivity map (spatial resolution 30 arc-seconds). Erosivity classes correspond to quantiles. Map generated with ESRI ArcGIS ver. 10.4 (http://www.esri.com); (**b**) number and cumulative percentage of GloREDa stations grouped by erosivity; **(c)** mean erosivity by continent; **(d)** mean erosivity by climate zone.

The number of GloREDa stations varied greatly among continents (Fig. 1). Europe had the largest contribution to the dataset, with 1,725 stations (48% of total), while South America had the lowest number of stations (141 stations or ~4% of total). Africa has very low density of GloREDa stations (5% of the total). In North America and the Caribbean, we collected erosivity values from 146 stations located in 6 countries (Unites States, Canada, Mexico, Cuba, Jamaica and Costa Rica). Finally, Asia and the Middle East were well represented in GloREDa, with 1,220 stations (34% of the total) distributed in 10 countries including the Siberian part of the Russian Federation (Fig. 1b). The geographic distribution within each continent also differed substantially. For instance, stations in Europe, Oceania and North America covered most of the territory, while those in Africa and South America were largely clustered. However, the stations are well distributed among different erosivity classes (Fig. 2b).

### Global erosivity map

The Gaussian Process Regression (GPR) model used to interpolate the erosivity (R-factor) point values to a map showed a good performance for the cross-validation dataset [R^2^ = 0.722, RMSE (Root Mean Square Error) = 1,629 MJ mm ha^−1^ h^−1^ yr^−1^]. The annual global erosivity map (Fig. 2) is presented at 30 arc-seconds (~1 km) spatial resolution and subdivided in 10 erosivity classes corresponding to the quantiles. The mean of the global R-factor map is 2,190 MJ mm ha^−1^ h^−1^ yr^−1^ with high variability as expressed by the standard deviation of 2,974 MJ mm ha^−1^ h^−1^ yr^−1^. The median (50^th^ percentile) of the global erosivity map is 1,150 MJ mm ha^−1^ h^−1^ yr^−1^ while 20% of the erosivity values (20^th^ percentile) are lower than 200 MJ mm ha^−1^ h^−1^ yr^−1^ and the highest 20% (80^th^ percentile) are higher than 5,200 MJ mm ha^−1^ h^−1^ yr^−1^ (Fig. 2). According to the global erosivity map, the highest values are located in south-eastern Asia (Cambodia, Indonesia, Malaysia, the Philippines and Bangladesh), Central Africa (Congo and Cameroon), South America (Brazil, Colombia and Peru), Central America and the Caribbean. The lowest erosivity is mainly located in Siberia, the Middle East, Northern Africa, Canada and Northern Europe. The Polar Regions have been masked out in the global erosivity map.

We found that the spatial patterns of the highest erosivity values (Fig. 2) are coincident with the corresponding patterns of extreme rainfall events reported by Zipser *et al*.^14^. Zipser *et al*.^14^ defined intense storms based on the convective vertical velocity of rain. The authors compiled a 7-year period (1998–2004) database of intense storms and they generated global maps of extreme rainstorm events based on lightening flash, brightness, temperature and noise. According to their study the highest frequency of extreme rainfall events (similar to high annual erosivity values) occurs in the central part of Latin America, Gulf of Mexico, central and western Africa, Madagascar, south-eastern Asia (mainly Bangladesh, south China), Indonesia and North Australia.

### Continental assessments

At the continental level, South America experiences the highest mean R-factor with 5,874 MJ mm ha^−1^ h^−1^ yr^−1^, followed by Africa (3,053 MJ mm ha^−1^ h^−1^ yr^−1^), Asia and the Middle East (1,487 MJ mm ha^−1^ h^−1^ yr^−1^). In Oceania, the mean R-factor was estimated at 1,675 MJ mm ha^−1^ h^−1^ yr^−1^(Fig. 2c).

Africa exhibits the highest erosivity estimates at the country level; Mauritius and Comoros have the highest worldwide mean annual erosivity values with an R-factor close to 20,000 MJ mm ha^−1^ h^−1^ yr^−1^. In Western Africa (Liberia, Sierra Leone and Equatorial Guinea), Central Africa (D.R of Congo, Republic of Congo and Cameroon) and Madagascar mean annual R-factor is higher than 7,000 MJ mm ha^−1^ h^−1^ yr^−1^. These patterns agree with those from other continental-scale assessments^15, 16^ which indicated highest erosivity values (>10,000 MJ mm ha^−1^ h^−1^ yr^−1^) along the Guinea coast of west and central Africa, the Congo basin and Madagascar. Ethiopia and South Africa have mean R-factor values close to 2,500 MJ mm ha^−1^ h^−1^ yr^−1^, but the spatial patterns are highly variable with the Ethiopian highlands having extremely high erosivity (>7,000 MJ mm ha^−1^ h^−1^ yr^−1^) while the lowlands have 3–4 times smaller values. The lowest mean R-factor, with values less than 115 MJ mm ha^−1^ h^−1^ yr^−1^, was estimated for Western Sahara, Libya and Egypt.

Within Asia, the Middle East has the lowest erosivity values, with a mean annual R-factor less than 220 MJ mm ha^−1^ h^−1^ yr^−1^ in Jordan, Saudi Arabia, Kuwait, Syria, Iran and Iraq (Fig. 2). China has a mean value of 1,600 MJ mm ha^−1^ h^−1^ yr^−1^, but exhibits high variability with zero erosivity in the arid north-west areas (Taklimakan desert), and extreme erosivity (>15,000) in the south-eastern coastal zones. Regional studies conducted by Zhu and Yu^17^ and Qin *et al*.^18^ show very similar spatial patters compared to our rainfall erosivity distribution in China. In Japan, the mean annual erosivity was estimated as 4,815 MJ mm ha^−1^ h^−1^ yr^−1^, a value close to the 5,130 MJ mm ha^−1^ h^−1^ yr^−1^ modelled by Shiono *et al*.^19^.

As expected the Siberian part of the Russian Federation and the former Union of Soviet Socialist Republics (Kazakhstan, Turkmenistan and Uzbekistan) have very low mean erosivity values (<250 MJ mm ha^−1^ h^−1^ yr^−1^) due their continental climate. On the contrary, Southeast Asia falls almost completely within the highest erosivity class (>7,400 MJ mm ha^−1^ h^−1^ yr^−1^), in agreement with national assessments for Peninsular Malaysia^20^. Their erosivity values, generated from pluviographic data range from 7,500 to 20,000 MJ mm ha^−1^ h^−1^ yr^−1^.

In South America, Chile has the lowest R-factor with a mean annual value 1,320 MJ mm ha^−1^ h^−1^ yr^−1^, followed by Argentina (2,232 MJ mm ha^−1^ h^−1^ yr^−1^). The rest of the South American countries have high mean erosivity values (>3,700 MJ mm ha^−1^ h^−1^ yr^−1^), with the highest ones in Brazil, Colombia and Ecuador (>7,000 MJ mm ha^−1^ h^−1^ yr^−1^). The erosivity gradient created by the Andes is clearly visible in the erosivity map. There were few national assessments on rainfall erosivity in south America^21–24^. Most of the data used for these studies have been used as input for GloREDa and their spatial patterns are in broad agreement to ours.

In North America and the Caribbean, the mean R-factor is 1,409 MJ mm ha^−1^ h^−1^ yr^−1^ with very low values in Canada and the Northern part of the United States, and extremely high values (>8,000 MJ mm ha^−1^ h^−1^ yr^−1^) along the Gulf of Mexico and the Caribbean countries. The erosivity map for the United States^25^ also shows high values along the Gulf of Mexico and southern Florida (>8,500 MJ mm ha^−1^ h^−1^ yr^−1^), while overall low values are observed in the Midwestern region (<690 MJ mm ha^−1^ h^−1^ yr^−1^).

In Australia, the mean R-factor is 1,535 MJ mm ha^−1^ h^−1^ yr^−1^ close to 1,767 MJ mm ha^−1^ h^−1^ yr^−1^ estimated by Teng *et al*.^26^ based on 11 years (2002–2012) satellite derived Tropical Rainfall Measuring Mission data. In terms of spatial patterns, Teng *et al*.^26^ also found maximum erosivity values along the northern and eastern coastal areas (>8,000 MJ mm ha^−1^ h^−1^ yr^−1^), which decreased towards the south-central region (<300 MJ mm ha^−1^ h^−1^ yr^−1^). In New Zealand, the high erosivity values (>4,000 MJ mm ha^−1^ h^−1^ yr^−1^) occur on the west coast of the South Island and decrease towards the east similar to the patterns observed by Klik *et al*.^27^ based on 35 weather stations.

The mean erosivity value for Europe was 488 MJ mm ha^−1^ h^−1^ yr^−1^, which is much lower than the one estimated by Panagos *et al*.^28^ for the European Union (722 MJ mm ha^−1^ h^−1^ yr^−1^). This is due to the inclusion of European Russia, Ukraine (422 MJ mm ha^−1^ h^−1^ yr^−1^) and Belarus (365 MJ mm ha^−1^ h^−1^ yr^−1^), all of which have low values compared to the other European countries.

### Analysis by Climate zones

**Figure 3:**
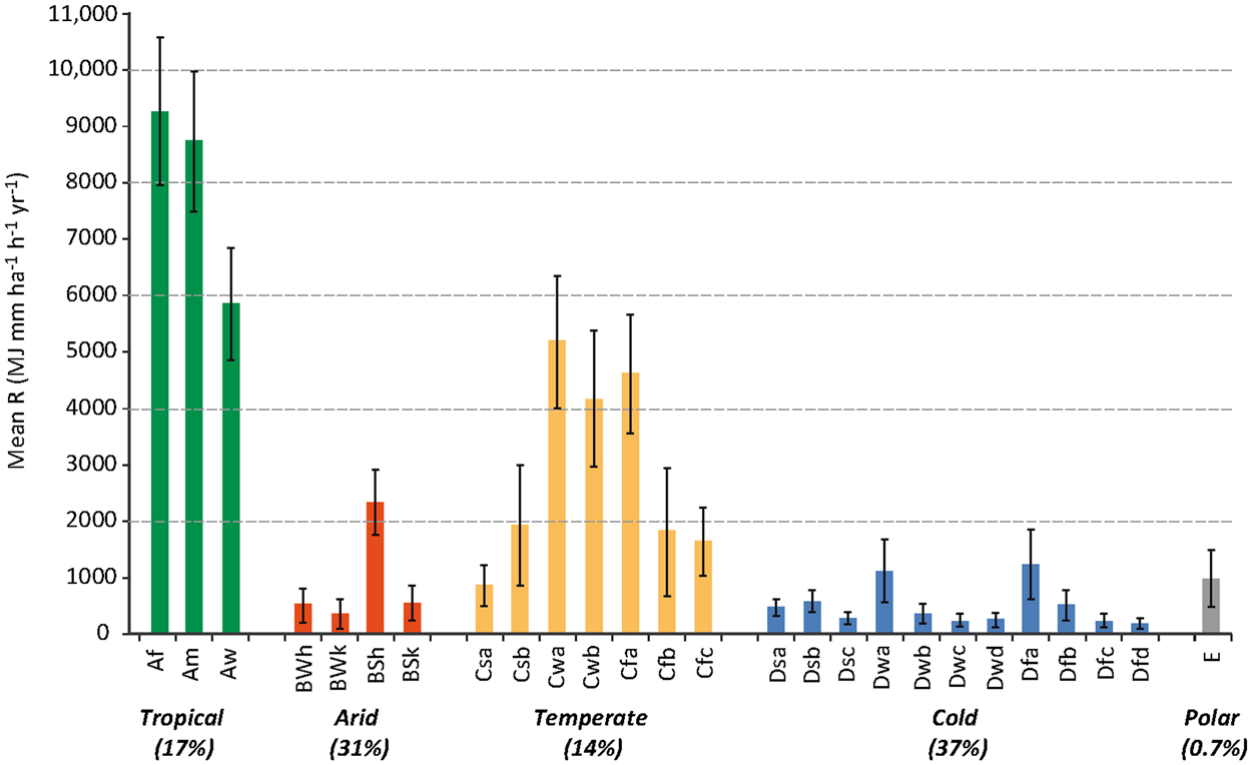
R-factor descriptive statistics per Kopper-Geiger climate type. Colour bars are the mean values per climate zone. Error bars represent the standard deviation. Percentages below each main climate category represent its proportion within the study area. Climate zones: Af (tropical rainforest), Am (tropical monsoon), Aw (tropical savannah), BWh (hot desert), BWk (cold desert), BSh (hot steppe), BSk (cold steppe), Csa (dry hot summer), Csb (dry warm summer), Cwa (subtropical dry winter), Cwb (dry winter and dry summer), Cfa (temperate without dry season and hot summer), Cfb (temperate without dry season and warm summer), Cfc (temperate without dry season and cold summer), DSa (cold and dry hot summer), Dsb (cold and dry warm summer), Dsc (cold and dry cold summer), Dwa (cold and dry winter, and hot summer), Dwb (cold and dry winter, and warm summer), Dwc (cold and dry winter, and cold summer), Dwd (cold and dry winter, and very cold winter), Dfa (cold without dry season and hot summer), Dfb (cold without dry season and warm summer), Dfc (cold without dry season and cold summer), Dfd (cold without dry season and very cold winter), E (polar).

The global rainfall erosivity map was further analysed per climate zones. The updated world Kopper-Geiger climate classification^29^ is the most widely used and accepted climate map in the scientific community. As expected, tropical climate group showed the highest mean erosivity with 7,104 MJ mm ha^−1^ h^−1^ yr^−1^. Within this group the tropical rainforest (Af) and monsoon (Am) climatic types had the highest mean erosivity and the lowest variability (Fig. 3). Second highest mean erosivity (3,729.3 MJ mm ha^−1^ h^−1^ yr^−1^) occurs in the temperate climate group.

The humid temperate, and temperate with dry winter climate type (Cfa, Cwa), mainly present in the southeastern United States, eastern Australia and southeast China, have mean erosivity values higher than 4,600 MJ mm ha^−1^ h^−1^ yr^−1^. The Mediterranean (Csa, Csb) and the Oceanic (Cfb) climate zones have mean erosivity values lower than 2,000 MJ mm ha^−1^ h^−1^ yr^−1^ (Fig. 3).

The arid climate group has a relatively low mean erosivity (842 MJ mm ha^−1^ h^−1^ yr^−1^) characterised by the highest spatial variability (e.g. the Cold desert (BWk) type). In this group, the hot desert (BWh) has the largest spatial share (13.9% of global area) with low mean erosivity values (537 MJ mm ha^−1^ h^−1^ yr^−1^). The cold desert climate (BWk), characteristic of northwest China and large areas of Kazakhstan, Turkmenistan, Uzbekistan, North Chile and Argentina, has a very low mean erosivity of 362 MJ mm ha^−1^ h^−1^ yr^−1^. The hot steppe climate (BSh), which is a transition from hot dessert to the tropical group (mainly in Africa and India), had medium mean erosivity of 2,371 MJ mm ha^−1^ h^−1^ yr^−1^.

The cold climate group had the lowest mean erosivity, with 493 MJ mm ha^−1^ h^−1^ yr^−1^ whereof the subarctic or boreal climate type (Dsc, Dwc, Dfc), covering major areas of Scandinavia, Siberia and Canada, had minimum mean erosivity values (<285 MJ mm ha^−1^ h^−1^ yr^−1^). By comparison, the climate zones immediately north of hot continental summers (Dfb, Dwb) that cover most of central and eastern Europe, European Russia and the northern United States, have much higher mean erosivity values (526 MJ mm ha^−1^ h^−1^ yr^−1^). The polar areas, mainly located in the Alps, Pyrenees and part of the Tibetan plateau, have a mean erosivity of approximately 990 MJ mm ha^−1^ h^−1^ yr^−1^.

The greatest uncertainty of the global erosivity map is likely related to transition areas between different climatic zones. The different climatic conditions, which result in high variability of rainfall amount, duration, magnitude and intensity, is the main reason for different spatial patterns of erosivity between climatic zones. The standard deviation shows the variability inside the climatic zone (Fig. 3). Moreover, the seasonal variation of climatic conditions play an important role in rainfall erosivity variability.

## Discussion

The increasing availability of rainfall data with high temporal resolution, the growing computing power, and the development of sophisticated geostatistical models, enabled the development of a global rainfall erosivity dataset at 30 arc-seconds (~1 km) spatial resolution. We acknowledge that this achievement was only feasible through the scientific cooperation between scholars from all over the globe. The global erosivity map was possible thanks to the contribution of data providers (see the long list of meteorological services, organisations, and institutions in the Acknowledgements section), tested methodologies and geostatistical models suitable for such a scale.

### Comparison with past studies

Compared to previous works on global R-factor estimation^30–32^, our study presents a data-driven approach including measured hourly and sub-hourly data on rainfall intensity for erosivity assessment. In contrast previous global erosivity assessments by Nachtergaele *et al*.^30^ and Yang *et al*.^32^ that used annual rainfall to estimate erosivity. However, as demonstrated by past literature^13, 28, 33^ and by the erosivity density statistics, the relation between rainfall amount and erosivity is not straightforward and as shown by Naipal *et al*.^31^ lead to an overestimation of rainfall erosivity. As a consequence, Naipal *et al*.^31^, included a simple precipitation intensity index calibrated to high resolution R-factor data of the USA.

**Figure 4:**
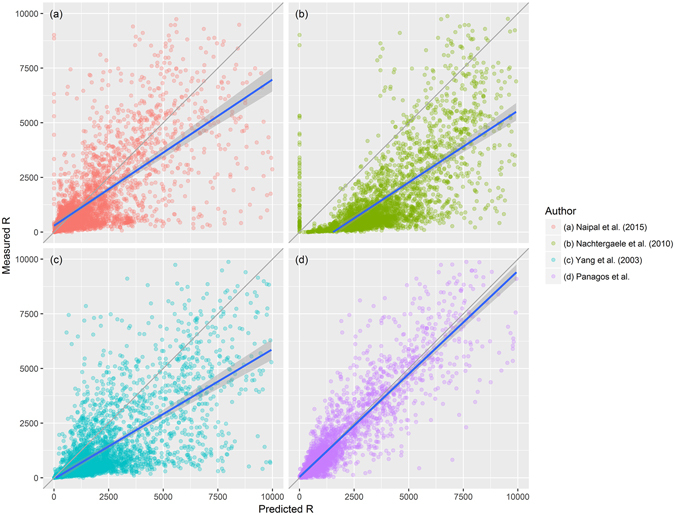
Comparison of predicted vs. measured R-factor values (values below 10,000 MJ mm^−1^ ha^−1^ yr^−1^) for the three previous and the presented global models. Grey line is the result of an optimal model (Intercept = 0 and regression coefficient = 1); Blue line is the regression result of each model; Grey zone is the 99% confidence interval for the coefficient.

In order to compare the three past erosivity maps^30–32^, and the current one, four Ordinary Least Square (OLS) models were fitted using the GloREDa measured values as an independent variable (Fig. 4). An optimal model should have a zero valued intercept and a regression coefficient as close as possible to one, thus matching the grey line of Fig. 4. Given these criteria our study outperforms the other models^30–32^ with a regression line very close to a coefficient of one. All the other models clearly show a significant bias (over-estimating the predictions) in either the slope or the intercept or both. Moreover, all the other models suffer from high variability as evidenced by the dispersion of the points cloud (Fig. 4).

Table 1 shows the comparison of the fitted models when the full measured range is considered. The intercept of the four models is shown in the first line as “B” column while the second line show the regression coefficients for each of the global models. The past studies^30–32^ have a high intercept bias with values deviating from 0 by more than 1,000, whereas the model proposed in this study has a much smaller value for the intercept (−204.2). The regression coefficient of the current study has a relatively small deviation from 1 (0.13), while the other models have a deviation between −0.27 and −0.85. Also in terms of R^2^ our model clearly outperforms all other models as it was fitted directly to the measured data (Table 1). Solely, the model from Nachtergaele *et al*.^30^. performs remarkably well compared to the models of Yang *et al*.^32^ and Naipal *et al*.^31^.

**Table 1:** Ordinary least squares coefficients (B) of global models for the assessment of rainfall erosivity (R-factor).

	*Naipal et al*.^31^			*Nachtergaele et al*. (*2010*)			*Yang et al*.^32^			*Panagos et al*.		
	*B*	*std. Error*	*p-value*	*B*	*std. Error*	*p-value*	*B*	*std. Error*	*p-value*	*B*	*std. Error*	*p-value*
Intercept	**1458.2**	279.3	<0.001	**−1149.0**	141.0	<0.001	**1223.8**	225.7	<0.001	**−204.2**	81.2	0.012
Regression coefficient	**0.15**	0.12	0.187	**0.73**	0.04	<0.001	**0.20**	0.08	.009	**1.13**	0.05	<0.001
Observations	3530			3530			3530			3530		
R^2^	**0.155**			**0.385**			**0.207**			**0.811**		

### Sources of uncertainty

Our inclusive approach to compile the maximum possible number of erosive events assumes that the data collected in one period is comparable with data from other periods. The inter-comparison of different time periods and the non-existence of other alternatives has been extensively discussed^28^, and has also been followed in similar data collection exercises such as the updated world map of the Koppen-Geiger climate classifications^29^. Obviously, this simplifying assumption is not valid at local scale, where observed trends in erosivity have been measured over a considerable period (e.g. 30 years). However, at the global scale, the inclusion of 3,625 stations smooths the potential bias due to the presence of trends and long-term temporal variability. Further, we preferred this inclusive approach over the alternative of selecting a common measurement period, since the latter would significantly reduce the station density entailing a reduction of the spatial prediction accuracy.

Past studies recommended a minimum period of 22-years for calculating long term R-factor, while 10-years measurements may lead to under or over-estimation^25, 34, 35^. As 12% of GloREDa includes stations with less than 10-years measurements, we recognize that this may cause a bias due to the temporal variation of R-factor. This bias, even if we are convinced that it is limited, should be considered.

Other uncertainties are related to the methodological approach such as the a) discrepancies in case of using a different algorithm for rainfall energy estimation (>97.7% of the calculated R-factor stations are based on the original RUSLE equation); b) the application of temporal resolution calibration factors found in European Union to the global dataset; c) the under-representativeness of measured R-factor data in highlands (11.6% of the total dataset is located in areas > 1000 m a.s.l. while the respective area amounts up to 20%), tropical and arid areas and d) the Gaussian Process Regression interpolation model. With regard to the first point the algorithm for unit rainfall energy (Equation 2) may underestimate the rainfall erosivity for instance in high erosive regions (e.g. Ethiopian highlands) due to large drop sizes^36^. The effect of the above listed uncertainties is considered not significant for the objectives of this product which is the model application at global scale. However, we propose that further improvements can be implemented in regional studies taking into account the best suitable algorithm for rainfall unit energy estimation, using more regional-based calibration factors to account for time resolution discrepancies and having a more representative (in terms of climate and topography) pool of stations with measured R-factor data.

### Implications of global erosivity map and data availability

The new global erosivity map is proposed for global and continental assessments of soil erosion by water, flood risk and natural hazard prevention. Therefore, the aim of the global erosivity dataset is not to challenge other local (or national) erosivity maps, developed from local data with better quality that may not have been available for the present study. Nevertheless, our global R-factor map can potentially cover gaps, where erosivity has not been estimated (due to lack of data), or where it has been calculated solely from rainfall amounts.

Current global estimates on soil erosion by water are very uncertain ranging over one order of magnitude from ca. 20 to over 200 Pg yr^−1^. More accurate global predictions of the rill and interrill soil erosion rates can only be achieved when the RUSLE rainfall erosivity factor is thoroughly computed. In this study, we present a robust global rainfall-runoff erosivity factor to measure the erosive potential of rainfall, which is a key input in soil erosion prediction models. The global erosivity map represents the first assessment that is solely based on the original RUSLE approach using sub-hourly measured rainfall data for 3,625 stations, providing a solid and harmonised basis for a robust spatial interpolation results. Our results show new insights into the global geography of rainfall erosivity and the global erosivity map which will be publicly available can be employed by other research groups to perform national, continental and global soil erosion modelling.

The GloREDa can be considered as an important step to bring together a large group of scientists to advance your understanding of large scale patterns related to land degradation, facing the current policy challenges and demands by the Food and Agriculture Organization (FAO), the Intergovernmental Platform on Biodiversity and Ecosystem Services (IPBES), and the United Nations Convention to Combat Desertification (UNCCD).

The Global erosivity map (GeoTIFF format) at 30 arc-seconds (~1 km) resolution is available for free download in the European Soil Data Centre (ESDAC) website at http://esdac.jrc.ec.europa.eu. The calculated erosivity values per station in GloREDa will become available in the future pending on the agreed copyright issues with data providers.

## Methods

The generation of the global erosivity map involved the following steps: a) the collection of high-temporal resolution rainfall data; b) the calculation of the erosivity factor (R-factor) for each rainfall station; c) the normalisation of R-factor values calculated with rainfall data collected at different time steps (1 min to 60 min), and d) the spatial interpolation of the R-factor point values.

### Collection of data

At global scale, this is the first time a data collection of observed (measured) high temporal resolution rainfall data takes place. The collection of high temporal resolution rainfall data from the maximum possible number of countries was considered necessary in order to have a representative sample across climatic and geographic gradients.

The Global Rainfall Erosivity Database (GloREDa) was compiled based on the following three components:

- Rainfall Erosivity database at European Scale (**REDES**) which includes 1,675 rainfall stations in the European Union and Switzerland (28 countries in total). REDES has been the data source for the erosivity map of Europe^28^ and was updated in 2015 to estimate monthly erosivity in Europe^13, 33^.
- A global data collection of both high resolution rainfall data and calculated erosivity values based on high temporal resolution rainfall data (Table 2). The data collection yielded 1,865 additional rainfall stations (around 52% of the total stations in GloREDa) from 23 countries outside of Europe. For the majority of the stations (located in China, Japan, India, Kuwait, Israel, Turkey, the United States of America, Mexico, Costa Rica, Jamaica, South Africa and Suriname) the calculation of the rainfall erosivity, based on high temporal resolution data, was performed for the first time (the calculation of rainfall erosivity, named as R-factor, is presented in the homonymous sub-section). For other regions, we used published erosivity data that were based on high temporal resolution rainfall datasets. This included countries such as Australia^37^, New Zealand^27^, South Korea^38^, Iran^39^, Malaysia^40^, Colombia^21^, Brazil^22^, Chile^23^, Mauritius^41^ and Algeria^42^.

**Table 2:** Overview of the high resolution rainfall data used to estimate global rainfall erosivity. In addition, erosivity information of 85 stations from 13 countries found in the literature^24, 43–56^ was included in the global map (not shown in the table).

Country		No. of Stations	(Main) Period Covered	Years per station (average)	(Main) Temporal resolution of rainfall data	Source of high temporal resolution rainfall data
AT	Austria	84	1995–2010	21	27 stations: 10Min; 57 stations: 15Min	Hydrographic offices of Upper Austria, Lower Austria, Burgenland, Styria, Salzburg, Carinthia, Vorarlberg and Tyrol.
AU	Australia	167	1961–2000	40	6 Min	Bureau of Meteorology in Australia; Yu *et al*.^37^
BE	Belgium - Flanders	20	2004–2013	10	30 Min	Flemish Environmental Agency (VMM),
BE	Belgium - Wallonia	29	2004–2013	10	60 Min	Service Public de Wallonie
BG	Bulgaria	84	1951–1976	26	30 Min	Rousseva *et al*.^57^
BR	Brazil	87	1986–2008	19	49 stations: 5 Min; 38 stations: 10 Min	Oliveira *et al*.^22^ and updated in 05/2016
CH	Switzerland	71	1988–2010	22	10 Min	MeteoSwiss
CL	Chile	18	1976–1995	17	15 Min	General Directorate of Water Resources (DGA), Government of Chile
CN	China	387	1998–2012	14	60 Min	National Meteorological Information Center of the China Meteorological Administration
CO	Colombia	6	1987–1996	10	5 Min	Centro Nacional de Investigaciones de Café - Cenicafé
CR	Costa Rica	5	2011–2015	6	30 Min	University of Costa Rica, Costa Rica
CY	Cyprus	35	1974–2013	39	30 Min	Cyprus Department of Meteorology
CZ	Czech Republic	32	1961–1999	35	30 Min	Research Institute for Soil and Water Conservation (Czech Republic)
DE	Germany	148	1996–2013	18	60 Min	Deutscher Wetterdienst (DWD)
DK	Denmark	30	1988–2012	15	60 Min	Danish Meteorological Institute (DMI), Aarhus University
DZ	Algeria	120	1977–2004	24	15 Min	National Agency of Hydraulic Resources, Algeria
EE	Estonia	21	2007–2013	7	60 Min	Estonian Environment Agency
ES	Spain	146	2002–2013	12	24 stations: 10 Min; 104 stations: 15 Min; 18 stations 30 MIN	Regional water agencies
FI	Finland	64	2007–2013	7	60 Min	Finnish Climate Service Centre (FMI)
FR	France	81	2004–2013	10	60 Min	Météo-France DP/SERV/FDP
GR	Greece	80	1974–1997	30	30 Min	Hydroskopio
HR	Croatia	42	1961–2012	40	10 Min	Croatian Meteo & Hydrological Service
HU	Hungary	30	1998–2013	16	10 Min	Hungarian Meteorological Service
IE	Ireland	13	1950–2010	56	60 Min	Met Éireann – The Irish National Meteorological Service
IL	Israel	61	1998–2015	17	30 Min	Israel Meteorological Service
IN	India	247	2007–2015	7	60 Min	India Meteorological Department, Ministry of Earth Sciences
IR	Iran	70	1984–2004	21	10 Min	Iranian Meteorological Organization
IT	Italy	251	2002–2011	10	30 Min	Regional meteorological services, Regional agencies for environmental protection (ARPA)
JM	Jamaica	9	2003–2014	12	2 Min	Meteorological service Jamaica
JP	Japan	55	2006–2015	10	60 Min	Japan Meteorological Agency (JMA)
KR	South Korea	75	1998–2015	18	10 Min	Korea Meteorological Administration (KMA)
KW	Kuwait	15	2007–2015	9	60 Min	Department of Meteorology, Directorate General of Civil Aviation, State of Kuwait
LT	Lithuania	3	1992–2007	16	30 Min	Lithuanian Agriculture and Forestry Research Centre
LU	Luxembourg	16	2000–2013	11	60 Min	Agrarmeteorologisches Messnetz
LV	Latvia	4	2007–2013	7	60 Min	Latvian Environment, Geology and Meteorology Centre
MU	Mauritius	5	2005–2008	5	6 Min	Mauritius Meteorological Services (MMS)
MX	Mexico	15	2006–2014	9	60 Min	CONAGUA, Comisión Nacional Del Agua, Servicio Meteorológico Nacional, Mexico.
MY	Malaysia	2	1981–1998	18	10 Min	Yu *et al*.^40^
NL	Netherlands	32	1981–2010	24	60 Min	Royal Netherlands Meteorological Institute
NZ	New Zealand	35	2000–2012	12	10 Min	New Zealand Institute of Water and Atmospheric Research (NIWA)
PL	Poland	13	1961–1988	27	30 Min	Warsaw University of Life Sciences
PT	Portugal	41	2001–2012	11	60 Min	Agência Portuguesa do Ambiente
RO	Romania	60	2006–2013	8	10 Min	Meteorological Administration
RU	Russian Federation	218	1961–1983	23	30 Min	Lomonosov Moscow State University
SE	Sweden	73	1996–2013	18	60 Min	Swedish Meteorological and Hydrological Institute (SMHI)
SI	Slovenia	31	1999–2008	10	5 Min	Slovenian Environment Agency
SK	Slovakia	103	1971–1990	20	60 Min	Slovak Hydrometeorological Institute, Climatological service
SR	Suriname	11	1987–2010	25	60 Min	Meteorological organization of Suriname
TR	Turkey	160	2005–2014	10	1 Min	Ministry of Forest and Water Affairs General Directorate of Combating Desertification and Erosion
UK	United Kingdom	38	1993–2012	20	60 Min	NERC & UK Environ. Change Network(ECN), British Atmospheric Data Centre (BADC)
US	United States of America	92	2006–2016	11	5 Min	U.S. Climate Reference Network (USCRN), NOAA; Diamond *et al*.^58^
ZA	South Africa	5	2001–2005	5	5 Min	Nel and Summer^41^
**Total**		**3,540**
- A literature review was used to fill some important data gaps, mainly in Africa, where high temporal resolution rainfall data are scarce. As a result of this exercise, rainfall erosivity values for 85 stations (2.4% of the whole database) from 13 countries were inserted in GloREDa. These countries included Canada^43^, Argentina^24^, Cuba^44^, Cape Verde^45^, Cameroon^46^, Eritrea^47^, Ethiopia^48^, Kenya^49, 50^, Niger^51^, Nigeria^52, 53^, Rwanda^54^, Tenerife^55^ and Zambia^56^.

Summarizing, we collected high temporal resolution rainfall data for 3,540 stations (97.7% of the total GloREDa). The total number of observational years equals 59,380; this results in an average of 16.8 years of high temporal resolution rainfall data per station. As such, GloREDa is the most comprehensive global database including the largest possible number of stations with high temporal resolution rainfall data.

### Calculation of rainfall erosivity (R-factor)

Rainfall erosivity accounts for the combined effect of rainfall duration, magnitude and intensity. In addition, it is also necessary to take into account the frequency of erosive events over a longer time period. In this study, the original R-factor from the Revised Universal Soil Loss Equation (RUSLE)^25^ was used for the vast majority (>97.7%) of the rainfall stations included in GloREDa. Accordingly, the calculation of rainfall erosivity (EI_30_) of a single event was based on the following equation:

1 $$E{I}_{30}=(\sum _{r=1}^{k}{e}_{r}{v}_{r})\,{I}_{30}$$

where e_r_ is the unit rainfall energy (MJ ha^−1^ mm^−1^) and v_r_ the rainfall volume (mm) during the r^th^ time period of a rainfall event divided in k-parts. I_30_ is the maximum 30-minutes rainfall intensity (mm h^−1^). The unit rainfall energy (e_r_) is calculated for each time interval as follows^59^:

2 $${e}_{r}=0.29[1-0.72{e}^{(-0.05{i}_{r})}]$$

where i_r_ is the rainfall intensity during the time interval (mm h^−1^).

R is the average annual rainfall erosivity (MJ mm ha^−1^ h^−1^ yr^−1^):

3 $$R=\,\frac{{\sum }_{j=1}^{n}{\sum }_{k=1}^{{m}_{j}}{(E{I}_{30})}_{k}}{n}$$

where *n* is the number of years recorded, *m*_*j*_ is the number of erosive events during a given year *j* and *k* is the index of a single event with its corresponding erosivity *EI*_*30*_.

Equation (2) was developed by Brown and Foster^59^ as part of the Revised Universal Soil Loss Equation (RUSLE, Renard *et al*.^25^), and replaced the original equation of Wischmeier and Smith^35^ used in the Universal Soil Loss Equation (USLE). Equation (2) was further modified as part of RUSLE2^60^, but its use has been mostly limited to the United States. However, outside of the United States the RUSLE equation is still the most commonly used and we have therefore applied it for our erosivity calculations. A comparison of the two equations yields slightly higher erosivity values for the RUSLE2 equation^60^. A recent study showed that empirical rainfall kinetic energy relationships compare well to measurement when complete events were considered (R^2^ > 0.90). Nonetheless, future research will explore how our current GloREDa results compare with alternative functions for rainfall energy.

According to the RUSLE handbook^25^, the erosive rainfall events were computed based on the following criteria: (i) the cumulative rainfall of an event is greater than 12.7 mm, (ii) the event has at least one peak that is greater than 6.35 mm during a period of 15 min (or 12.7 mm during a period of 30 min) and, (iii)a rainfall accumulation of less than 1.27 mm during a period of six hours splits a longer storm period into two storms. The erosivity factor equations and the above mentioned criteria have been developed according to more than 10,000 plot-years of experiments. The R-factor for each station in GloREDa was calculated using the Rainfall Intensity Summarisation Tool (RIST) software developed by the United States Department of Agriculture (USDA).

### Calibration of different time resolutions

For such an extensive data collection, it is expected to have a variety in both i) the range of available data-years and ii) the time resolution of the data. According to the GloREDa statistics, 35.7% of the stations had rainfall data at very high resolution (<=15 min); ~25.7% had an intermediate resolution (30 minutes), while the remaining 38.6% had a resolution of 60 minutes. Due to this heterogeneity, a time step calibration of erosivity values was considered necessary.

We selected a 30-minute time resolution as an acceptable compromise between the coarse time resolution of 60 minutes and the higher ones (<=15 minutes). For the calibration, we have selected a pool of 86 rainfall stations as their data were available at multiple temporal resolutions and their geographical coverage is representing 14 countries covering a large climate gradient. The calibration process included three steps: a) The R-factor was calculated at the highest possible resolution (5, 10 or 15 minutes); b) The rainfall data were aggregated to coarser resolutions (30 and 60 minutes) and the corresponding R-factor was calculated at the coarser resolutions and c) The calibration factors was derived from the regression analysis of the R-factor calculations at the highest and coarser resolutions. Those calibration factors have been developed in the European study^33^ and were in agreement with range values provided in the literature^25, 60, 61^ which have been calculated in China, South Italy and U.S.A.

### Interpolation of GloREDa

Given the correlation between the R-factor and monthly climatic data^28^, a regression approach was used to infer the spatial global distribution of rainfall erosivity from a series of independent climatic covariates derived from the WorldClim database^62^. The covariates used included average monthly precipitation, average minimum and maximum monthly precipitation, average monthly temperature, precipitation of the wettest month, precipitation of the driest month, and precipitation seasonality, as defined in the WorldClim database (www.worldclim.org). These variables represent long term conditions based on the interpolation of observed data for the period 1960–1990^62^. As a result, our erosivity map represents long-term erosivity patterns. Elevation was not included in the model, as it was already used to estimate some of the WorldClim variables. We subsequently assembled a dataset where each location (i.e. rainfall station) had a mean R-factor (independent variable) as well as values for the climatic variables (independent variables), and used it as input for the interpolation.

We used Gaussian Process Regression (GPR)^63, 64^ due to the large number of support covariates, their potential collinearity, and the presence of non-linear relationships between the target variable (R-factor) and the covariates. GPR is a non-linear regression approach that can model non-linear relations by projecting the inputs into a higher dimensional space using basis functions, and by creating a regression model in said space. Considering the regression form ${\rm{y}}=f({\rm{x}})+{\mathscr{N}}(0,\,{\sigma }_{n}^{2})$ with $f({\rm{x}})={{\rm{x}}}^{T}{\bf{w}}$ (where *y* are the observed responses, *x* the covariates values vector, *f* a set of functions and **w** a vector of weights), the GPR uses a projection from the original input space into a feature space using kernel expansion so that f(x) can be rewritten as $f(x)=\varphi {({\bf{x}})}^{T}{\bf{w}}$ where *ϕ* is the kernel function.

In this study the Radial Basis Function (RBF) Gaussian kernel was used, which can be written as $K({\bf{x}},{\bf{x}}^{\prime} )=\exp (-\frac{\parallel {\bf{x}}-{\bf{x}}^{\prime} {\parallel }^{2}}{2{\sigma }^{2}})$ where *σ* is free tunable parameter. The Gaussian kernel is highly adaptable and commonly applied in machine learning^65^. The main advantages of GPR are that it can model very complex non-linear relations between covariates and the target variable, and directly model both average and variance estimation, thus providing information about prediction uncertainty. Moreover, GPR is resistant to the issues derived from collinearity among independent variables that can arise in other statistical models. Finally, the GPR has the advantage of adapting in local conditions (climatic) due to its non-parametric nature. In this study the GPR model performance was tested for both, a fitting, and a cross-validation dataset. The cross-validation was carried out by random sampling with 10% replacement of the original dataset used for validation.
